# Testicular choriocarcinoma with pelvic and pulmonary metastases: a case report

**DOI:** 10.3389/fonc.2024.1427341

**Published:** 2024-07-18

**Authors:** Xin Bai, Xiao H. Liu, Hai W. Liang, Yi S. Li, Biao F. Shan, Jian M. Tang

**Affiliations:** ^1^ The First Clinical Medical College, Lanzhou University, Lanzhou, Gansu, China; ^2^ Department of Radiation Oncology, The First Hospital of Lanzhou University, Lanzhou University, Lanzhou, Gansu, China

**Keywords:** testicular choriocarcinoma, pelvic metastases, lung metastases, genital examination, retroperitoneal mass

## Abstract

Testicular tumors represent a common form of solid tumor in young men, with choriocarcinoma of the testis being a rare, non-granulomatous germ cell tumor. It accounts for less than 0.3% of all testicular germ cell tumors. Pelvic and pulmonary metastases originating from testicular choriocarcinoma are exceptionally uncommon in men. This study describes a case of a 27-year-old male diagnosed with testicular choriocarcinoma, presenting initially with nausea, vomiting, and abdominal pain. Furthermore, this review encompasses cases of testiclar choriocarcinoma in individuals aged 30 years and below, both in China and internationally, over the past 20 years.

## Introduction

Choriocarcinoma, a malignant trophoblastic tumor, secretes human chorionic gonadotropin (β-HCG). Although the majority of choriocarcinomas develop in women following either normal or abnormal pregnancies, their occurrence in non-pregnant individuals is exceptionally rare (1). Testicular choriocarcinoma, in particular, is extremely uncommon, comprising less than 0.3% of all testicular germ cell tumors. Typically occurring as part of mixed germ cell tumors, pure choriocarcinoma is especially rare. Previous meta-analyses have determined the most common sites of metastasis to be the lungs, vagina, brain, liver, and kidneys, listed in descending order. Other less frequently involved sites include the pancreas, small and large intestines, bone marrow, and abdominal wall (2). This article examines a complex case of choriocarcinoma metastasizing to the lungs, retroperitoneum, and pelvis, presenting as abdominal pain and nausea.

## Clinical history and pathology

On 9 January 2023, a 25-year-old man reported pain in the abdomen and left lower back, accompanied by nausea and vomiting. No fever, chills, diarrhea, or blood in the stool were reported. Initially, the patient disregarded these symptoms. On 16 January, he sought treatment at another hospital where an ultrasound examination revealed solid lesions in the retroperitoneal area, likely neoplastic, causing compression of the left upper ureter, dilatation of the left ureter with pyelonephritis, salt crystals in the renal urethra, and no abnormalities in the right kidney. Suspecting ectopic pheochromocytoma, retrograde angiography and stenting of the left ureter were performed under local anesthesia on 16 January 2023, subsequently, the patient was discharged.

**Figure 1:**
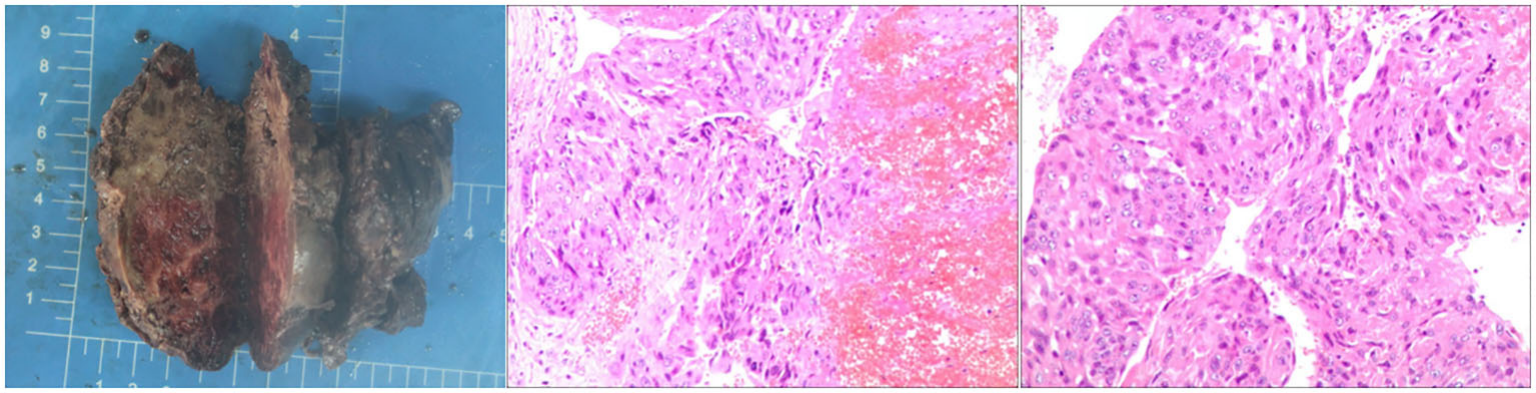
The post-op specimen measures 9x8x4.5 cm and shows grey-red to grey-brown inelastic tissue. Cross-section displays grey-red and grey-white areas with necrotic and hardened regions. Diagnosed as a malignant germ cell tumor (left retroperitoneal), consistent with choriocarcinoma of epithelial origin per immunohistochemical staining.

Subsequently, on 31 January, the patient experienced intermittent worsening of pain with nausea and vomiting, which intensified with eating and drinking. A subsequent CT scan of the entire abdomen at that medical facility revealed an irregular mass in the left retroperitoneum invading the ureter, abdominal segments, and abdominal aorta. On 3 February 2023, resection of the left retroperitoneal mass was conducted under general anesthesia. Postoperative evaluation identified a left retroperitoneal germ cell malignancy, and immunohistochemical analysis confirmed choriocarcinomac (**Figure 1**). Further ultrasound evaluation of the reproductive system detected a solid mass within the left testis, most likely a homologous germ cell tumor. Recommendations included further sequencing and follow-up treatment. Upon discharge, the physician advised a follow-up in three weeks, which would include removal of the intratesticular mass and 3-4 cycles of postoperative chemotherapy with a BEP (bleomycin, etoposide, and cisplatin) regimen.

On 22 February 2023, the patient reported increased abdominal pain. A subsequent CT scan displayed a soft tissue mass at the previous surgery site, indicating tumor recurrence. This recurrence affected multiple sites, including the nearby abdominal aorta, inferior vena cava, upper left ureter, left psoas muscle, and neighboring bowel. Consequently, the left kidney and upper ureter were observed to be dilated and exhibited hydrops. Additionally, the left ureter showed signs of stenting, with a curvature at the end. Further, some mildly enlarged mesenteric root lymph nodes were noted, along with suspicious small nodules in the mesenteric vessel wall in the pelvic region, necessitating regular monitoring. The patient developed hydronephrosis, leading the urology department to recommend replacing the ureteral stent. As the existing stent was deformed and could not accommodate the guidewire, a left nephrostomy and drainage were performed in 2023. Despite receiving symptomatic supportive treatment, the patient’s condition had not significantly improved by 29 February 2024 when he was admitted to our hospital.

The patient was admitted to the hospital with the following metrics: height of 176 cm, weight of 60 kg, body surface area of 1.76 cm², NRS score of 0, and a KPS score of 70. The general condition of the patient was characterized by normal growth, malnutrition, and facial pain, however, gait, posture, and mental state were normal, and cooperation during the examination was observed.

Past medical history indicated that the patient had been healthy, with no prior surgical interventions or trauma, no history of hepatitis or tuberculosis, no infectious diseases, no known exposures, no vaccinations, no drug allergies, and no blood transfusions. In terms of personal history, the patient was single, had not resided in infectious areas, and had abstained from smoking, alcohol, and substance abuse, with no exposure to industrial toxins, dust, or radiation, and no travel history. No genetic diseases were reported in the family history.

**Figure 2:**
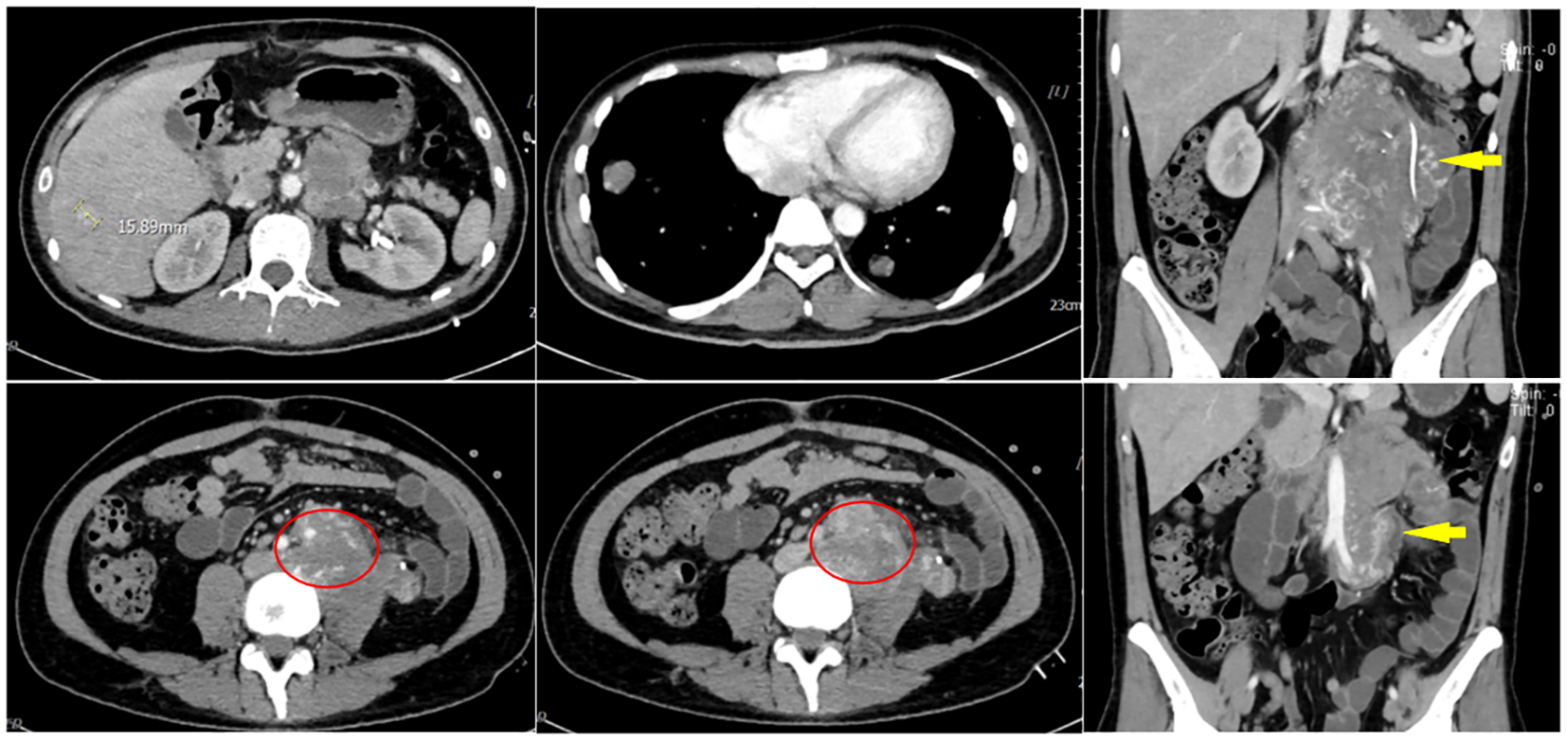
On March 1, 2023, an abdominal CT scan revealed irregular mixed-density masses in the retroperitoneum and left abdominal cavity, measuring 66 mm × 82 mm and 46 mm × 44 mm, respectively. These masses surrounded the abdominal aorta, extended below the bifurcation, and affected the left psoas major muscle and ureter definition. Heterogeneous enhancement in the mass with migrating vascular shadows was observed, along with multiple lymph nodes in the left abdominal cavity and a nodular omentum. Additionally, multiple intensifying nodules were noted in the right hepatic lobe during the arterial phase, with isodensity in the portal and delayed phases. No fluid was present in the abdominal cavity, but enlargement of retroperitoneal lymph nodes and multiple nodules in both lungs were suspected to be metastatic.

Laboratory results revealed an erythrocyte count of 2.88×10¹²/L (outside the reference range of 130-175), and a hemoglobin level of 86g/L (below the reference range of 130-175g/L). The β-HCG level was over 10,000mIU/ml (exceeding the reference range of 0-2.60 mIU/ml), and the LDH stood at 518U/L (above the reference range of 120-250U/L), indicating elevated levels. Other noted abnormalities included increased levels of ferritin, total and direct bilirubin, alkaline phosphatase, aspartate aminotransferase, alanine aminotransferase, and glutamyl aminotransferase. Both fecal and urinary occult blood tests yielded positive results. On March 1, 2023, an abdominal CT scan was performed on the patient, revealing irregular mixed-density masses in the retroperitoneum and left abdominal cavity, measuring 66 mm × 82 mm and 46 mm × 44 mm, respectively. These masses surrounded the abdominal aorta, extends below the bifurcation, and affected the left psoas major muscle and ureter definition. Heterogeneous enhancement in the mass with migrating vascular shadows was observed, along with multiple lymph nodes in the left abdominal cavity and a nodular omentum. Additionally, multiple intensifying nodules were noted in the right hepatic lobe during the arterial phase, with isodensity in the portal and delayed phases. No fluid was present in the abdominal cavity, but enlargement of retroperitoneal lymph nodes and multiple nodules in both lungs were suspected to be metastatic (**Figure 2**).

**Figure 3:**
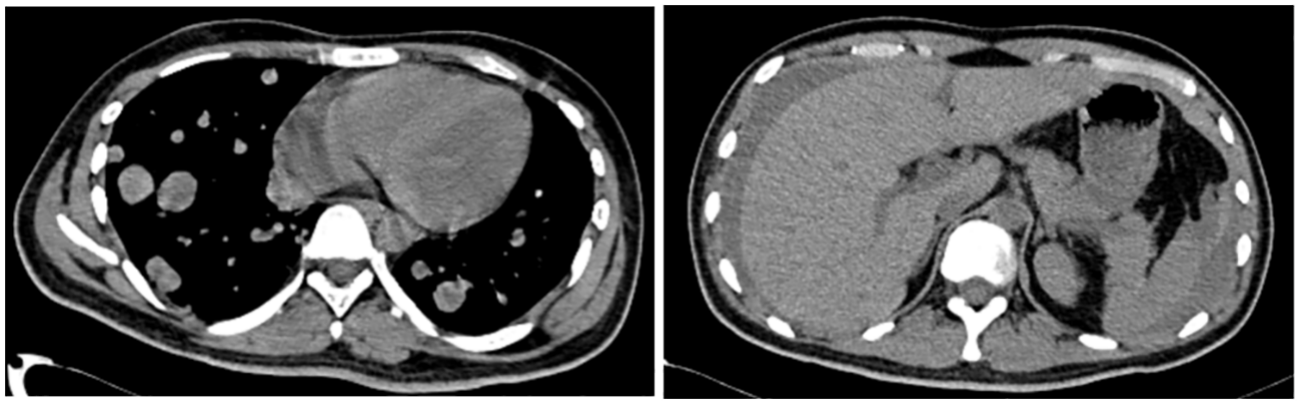
A full abdominal CT scan on 2 March revealed: 1) a new hyperdense shadow in the abdominal cavity indicative of potential hemorrhage, necessitating clinical correlation; 2) a notably increased number of lung nodules compared to previous scans, and 3) a fresh abdominopelvic effusion.

The patient’s initial condition was poor, characterized by abdominal and back pain. Following the administration of hepatoprotective and antiemetic medications, symptoms of pain were alleviated. However, at 20:10 on 1 March, the patient suffered a sudden altered state of consciousness, displaying dilated pupils at 5 mm and diminished light responsiveness. Within two minutes, both consciousness and pupil size reduced to 3 mm, with a fading light reflex absent any obvious pathology signs. Through anti-infective and supportive therapies, continuous cardiac monitoring, and consultations with multiple specialists, the patient’s condition gradually stabilized. To investigate the cause of the abdominal pain, a CT scan of the whole abdomen on 2 March revealed (**Figure 3**): 1) a new hyperdense shadow in the abdominal cavity indicative of potential hemorrhage, necessitating clinical correlation; 2) a notably increased number of lung nodules compared to previous scans, and 3) a fresh abdominopelvic effusion. A diagnostic laparotomy was performed, yielding non-clotting blood that confirmed a ruptured retroperitoneal mass with associated bleeding. Treatment included a red blood cell transfusion and continued supportive care. The medical team informed the family of the severe risks including shock and respiratory failure due to tumor rupture and hemorrhage. They opted against transfer to the ICU or extubation. After consultations with interventional and general surgeons, immediate intervention was deemed risky and ineffective. The risks of surgical hemostasis included potential respiratory and circulatory failure during the procedure. The family remained undecided upon learning of these risks and the ongoing need for treatment, leading to a decision to continue with symptomatic supportive care. on 3 March, the family declined surgery, fluids, endotracheal intubation for potential respiratory or circulatory failure, mechanical ventilation, and all resuscitative efforts, requesting solely pain management and signed a “refusal of treatment” notice. Thus, interventions were confined to managing pain. At 20; 44 on 6 March, the patient ceased to breathe spontaneously, aortic pulsation was absent, pupils were dilated and unresponsive, and the ECG registered as flat; he was pronounced clinically dead.

## Discussion

Imaging features of testicular choriocarcinoma are insufficiently distinct to differentiate it from other germ cell tumors. Diagnosis is challenging and typically relies on pathological confirmation during surgical resection. Treatment response monitoring is conducted through serum levels of beta-human chorionic gonadotropin (beta-HCG). According to the International Germ Cell Carcinoma Collaborative Group, beta-HCG levels exceeding 50,000 IU/L are indicative of a poor prognosis. While testicular cancer generally has high cure rates, with five-year survival rates reaching 95 percent, choriocarcinoma responds poorly to chemotherapy. Patients often exhibit severe symptoms and demonstrate a poor response to standard chemotherapy regimens, including three to four cycles of BEP (bleomycin, etoposide, and cisplatin). In cases of recurrence, salvage chemotherapy with vincristine and ifosfamide may decrease tumor burden, but some patients may require palliative care (3). As fewer patients are included in **Table 1** compared to the statistical analysis, it is clear that survival rates are significantly lower for patients treated solely with surgery, while combining surgery with other therapies markedly improves survival duration (13, 14).

**Table 1:** Clinical data of gastrointestinal bleeding caused by metastatic testicular choriocarcinoma.

Case	Year	Age	First symptoms	Primary site of metastasis	Treatment	Ending
1 (4)	2009	24	Painless lump in left testicle	Lungs, brain, liver, intestines	Surgical+chemotherapy+Radiotherapy	live
2 (5)	2009	17	Testicular lumps	Lungs, liver, duodenum	Surgical+chemotherapy	live
3 (6)	2011	25	Dyspnoea, black stools, weight loss	Lungs, scrotum, skin	Surgical	Death
4 (7)	2012	24	Black stools, palpitations	lungs	Surgical+chemotherapy+Radiotherapy	Death
5 (8)	2012	24	tachycardia	Lungs, kidneys, scrotum, stomach	Surgical+chemotherapy	live
6 (5)	2015	18	Black stools, drowsiness, dizziness	Brain, lungs, lymph nodes, stomach	Surgical+chemotherapy	live
7 (9)	2019	30	Shortness of breath, chest pain	Lungs, duodenum, stomach	Symptomatic supportive therapy	live
8 (3)	2020	17	shock	Brain, duodenum, scrotum	Surgical+chemotherapy	live
9 (10)	2022	28	dark stool	Lungs, liver, jejunum	Surgical+chemotherapy	live
10 (11)	2022	20	Dyspnoea, black stools	lungs	chemotherapy	live
11 (12)	2023	27	Enlargement of the right scrotum	Lungs, Bones	Surgical+chemotherapy	live

Death refers to death during hospitalization.

In this article, the initial out-of-hospital evaluation neglected to assess the reproductive system or conduct HCG testing, leading to diagnostic delays. Testicular choriocarcinoma progresses rapidly, yet the outpatient facility mistook it for a pheochromocytoma without adequately examining the retroperitoneal mass. This oversight delayed treatment and excluded ultrasonography of the reproductive system and HCG monitoring. Conversely, immediate initiation of cisplatin-based chemotherapy, bypassing pre-surgery retroperitoneal examination, could improve patient survival. Tumor rupture and bleeding were primary causes of mortality, where radiotherapy could assist in controlling bleeding.

This case highlights the critical need for comprehensive history taking, systematic examination, and thorough laboratory testing in patients presenting with abdominal pain and nausea. Early and accurate diagnosis of testicular choriocarcinoma is crucial, as delayed detection increases mortality rates, prolongs hospital stays, and raises healthcare expenses. Therefore, an initial assessment of the reproductive system is essential. Preventing tumor rupture and hemorrhage, and enhancing the overall condition of the patient, are crucial for effective anti-tumor treatment. Early identification, prompt intervention, and precise diagnosis are essential for improving patient outcomes.

Examination of the reproductive system should be integrated into the systematic evaluation of patients, irrespective of apparent clinical correlation, to ensure a comprehensive approach. Early and prompt diagnosis and treatment of testicular choriocarcinoma are vital for enhancing patient survival rates. While the approach in this case was not without flaws, it serves as a critical reminder of the importance of early detection and intervention in cancer care.

## Data availability statement

The original contributions presented in the study are included in the article/supplementary material. Further inquiries can be directed to the corresponding author.

## Ethics statement

The studies involving humans were approved by Ethics Committee of the First Hospital of Lanzhou University. The studies were conducted in accordance with the local legislation and institutional requirements. Written informed consent for participation in this study was provided by the participants’ legal guardians/next of kin. Written informed consent was obtained from the individual(s) for the publication of any potentially identifiable images or data included in this article.

## Author contributions

XB: Conceptualization, Data curation, Formal analysis, Funding acquisition, Investigation, Methodology, Project administration, Resources, Software, Supervision, Validation, Visualization, Writing – original draft, Writing – review & editing. XL: Conceptualization, Data curation, Investigation, Software, Writing – review & editing. HL: Conceptualization, Data curation, Investigation, Methodology, Writing – original draft. YL: Conceptualization, Data curation, Investigation, Methodology, Writing – original draft. BS: Formal Analysis, Investigation, Methodology, Validation, Writing – review & editing. JT: Conceptualization, Data curation, Formal analysis, Funding acquisition, Investigation, Methodology, Project administration, Resources, Software, Supervision, Validation, Visualization, Writing – review & editing, Writing – original draft.
